# Tumour necrosis factor (TNF) and interleukin-1 (IL-1) induce muscle proteolysis through different mechanisms

**DOI:** 10.1155/S0962935192000371

**Published:** 1992

**Authors:** Oded Zamir, Per-Olof Hasselgren, Takashi Higashiguchi, Janice A. Frederick, Josef E. Fischer

**Affiliations:** Department of Surgery University of Cincinnati Medical Centre 231 Bethesda Avenue Cincinnati Ohio 45267-0558 USA

## Abstract

The purpose of this study was to test the hypothesis that muscle proteolysis induced by TNF or IL-1 is mediated by glucocorticoids. Rats were treated with 300 μg kg^−1^ of recombinant human preparations of IL-1α (rIL-1α) or TNFα (rTNFα) divided into three equal intraperitoneal doses given over 16 h. Two hours before each cytokine injection, rats were given 5 mg kg^−1^ of the glucocorticoid receptor blocker mifepristone RU 38486, by gavage or were gavaged with the vehicle. Eighteen hours after the first cytokine injection, total and myofibrillar protein breakdown rates were determined in incubated extensor digitorum longus muscles as release of tyrosine and 3-methylhistidine, respectively. Total and myofibrillar proteolytic rates were increased following injection of rIL-1α or rTNFα. Proteolysis induced by rIL-1α was not altered by treatment with RU 38486. In contrast, the glucocorticoid receptor blocker inhibited the proteolytic effect of rTNFα. The results suggest that the proteolytic effect of TNF is mediated by glucocorticoids and that IL-1 induces muscle proteolysis through a glucocorticoid independent pathway.


Mediators of Inflammation, 1, 247–250 (1992)

### Tumour necrosis factor (TNF) and interleukin-1 (IL-1) induce muscle proteolysis through different mechanisms

Oded Zamir, Per-Olof Hasselgren,[CA] Takashi Higashiguchi, Janice A. Frederick and Josef E. Fischer

Department of Surgery, University of Cincinnati Medical Centre, 231 Bethesda Avenue, Cincinnati, Ohio, 45267-0558, USA

[CA] Corresponding Author

**The purpose of this study was to test the hypothesis that muscle proteolysis induced by TNF or IL-1 is mediated by glucocorticoids. Rats were treated with 300 $\mu$g kg$^{-1}$ of recombinant human preparations of IL-1α (rIL-1α) or TNFα (rTNFα) divided into three equal intraperitoneal doses given over 16 h. Two hours before each cytokine injection, rats were given 5 mg kg$^{-1}$ of the glucocorticoid receptor blocker mifepristone RU 38486, by gavage or were gavaged with the vehicle. Eighteen hours after the first cytokine injection, total and myofibrillar protein breakdown rates were determined in incubated extensor digitorum longus muscles as release of tyrosine and 3-methylhistidine, respectively. Total and myofibrillar proteolytic rates were increased following injection of rIL-1α or rTNFα. Proteolysis induced by rIL-1α was not altered by treatment with RU 38486. In contrast, the glucocorticoid receptor blocker inhibited the proteolytic effect of rTNFα. The results suggest that the proteolytic effect of TNF is mediated by glucocorticoids and that IL-1 induces muscle proteolysis through a glucocorticoid independent pathway.**

**Key words:** Glucocorticoids; IL-1; Protein breakdown; Skeletal muscle; TNF.

### Introduction

Previous studies suggest that tumour necrosis factor (TNF) and interleukin-1 (IL-1) can stimulate muscle protein breakdown,[1-4] although conflicting results have been reported.[5] The mechanism by which TNF and IL-1 exert this metabolic effect is unknown. A direct effect on protein metabolism could not be verified when IL-1 or TNF was added to incubated muscles.[5-8] Because both cytokines can induce release of glucocorticoids[9-11] it has been suggested that the metabolic effects of these cytokines involve hormonal stimulation.[10,12] The purpose of this study was to test the hypothesis that muscle proteolysis induced by TNF or IL-1 is mediated by glucocorticoids. This was done by administering the cytokines to rats that had been treated with the glucocorticoid receptor antagonist RU 38486.[13]

### Materials and Methods

The study was performed using male Sprague–Dawley rats weighing 40–60 g (Zivic-Miller Laboratories, Inc., Zelienople, PA). The animals were housed in groups of three per cage at 22°C room temperature with a 12 h light/dark cycle, and were allowed to acclimatize for 3 days before experiments. Three series of experiments were performed. In the first series of experiments, groups of rats were treated with three intraperitoneal injections of human recombinant IL-1α (rIL-1α), 100 $\mu$g kg$^{-1}$ body weight each, at 16:00, 24:00 and 08:00 h. The cytokine (kindly provided by Hoffman-La Roche, Nutley, NJ) had a specific activity of $3 \times 10^8$ U mg$^{-1}$ protein (D10 assay) and contained less than 0.75 endotoxin units/mg protein (determined by Limulus lysate assay). The cytokine was diluted in phosphate buffered saline (PBS), pH 7.4, to a final concentration of 10 $\mu$g ml$^{-1}$. Other rats received corresponding intraperitoneal injections of PBS. Both cytokine and PBS treated rats were divided into two subgroups. Animals in one subgroup were treated with three doses (5 mg kg$^{-1}$ body weight each) of the glucocorticoid receptor antagonist RU 38486 (kindly provided by Roussel-Uclaf, Romainville, France) administered by gavage 2 h before each cytokine or PBS injection. RU 38486 was suspended in an aqueous solution of 0.20% polysorbate (Tween 80; Sigma Chemical Co., St. Louis, MO) and 0.25% carboxymethylcellulose (Sigma). Animals in the other subgroup received corresponding volumes of the vehicle by gavage.

In the second series of experiments, an identical protocol was employed except that human recombinant TNFα (rTNFα) was injected instead of rIL-1α. The rTNFα preparation (kindly provided by Knoll Pharmaceuticals, Whippany, NJ) had a specific activity of greater than $5 \times 10^6$ U mg$^{-1}$ protein (L929 cytotoxicity test) with an endotoxin content of less than 5 U mg$^{-1}$ protein (Limulus lysate assay).

© 1992 Rapid Communications of Oxford Ltd

Muscle protein breakdown rates were measured 2 h after the last cytokine or PBS injection. With rats under ether anaesthesia, the extensor digitorum longus muscles were dissected with their tendons intact. The muscles were tied by their tendons on stainless steel supports at resting length and incubated individually in 3 ml of oxygenated ($O_2$:$CO_2$, 95:5) Krebs-Henseleit bicarbonate buffer (pH 7.4) with 10 mM glucose in a shaking water bath at 37°C. After preincubation for 30 min, the muscles were incubated for 2 h in 3 ml of fresh oxygenated medium of the same composition as described above but also containing cycloheximide (0.5 mM) to prevent reutilization of amino acids released during proteolysis. Total and myofibrillar protein breakdown rates were determined as release of tyrosine and 3-methylhistidine (3-MH), respectively, taking changes in tissue levels of the amino acids during incubation into account, as described in detail previously.[14]

In the third series of experiments, we examined the effect of rIL-1α and rTNFα on plasma levels of corticosterone, the predominant glucocorticoid in rodents. rIL-1α or rTNFα was injected intraperitoneally at a dose of 100 $\mu g\ kg^{-1}$ body weight at 08:00 h. Control animals received corresponding volumes of PBS. Blood was collected into heparinized beakers following decapitation 30 min, 2 h, 4 h and 8 h after cytokine or PBS injection. Blood was centrifuged and the plasma was stored at −70°C until assayed. Corticosterone levels were determined by radioimmunoassay.[15]

Results are presented as mean ±SEM. For statistical comparison analysis of variance followed by Duncan's multiple range test was used. Differences were considered significant for $p < 0.05$.

### Results

Rats exhibited symptoms of lethargy, piloerection and loose stool following injection of rIL-1α or rTNFα, and these symptoms were slightly more prominent following rIL-1α than rTNFα administration. There was no mortality when rIL-1α or rTNFα was injected into rats that had not been treated with RU 38486. In the groups of rats that had been treated with RU 38486, mortality rates following rIL-1α and rTNFα were 33% (10/30) and 7% (2/30), respectively.

Total protein breakdown rate, expressed as tyrosine release, was increased by 46% and myofibrillar protein breakdown rate, expressed as 3-MH release, was increased more than two-fold following administration of rIL-1α (Fig. 1). Treatment with RU 38486 did not alter the effect of rIL-1α on total or myofibrillar protein breakdown rates (Fig. 1). The glucocorticoid receptor antagonist did not affect protein breakdown rates in control injected rats.

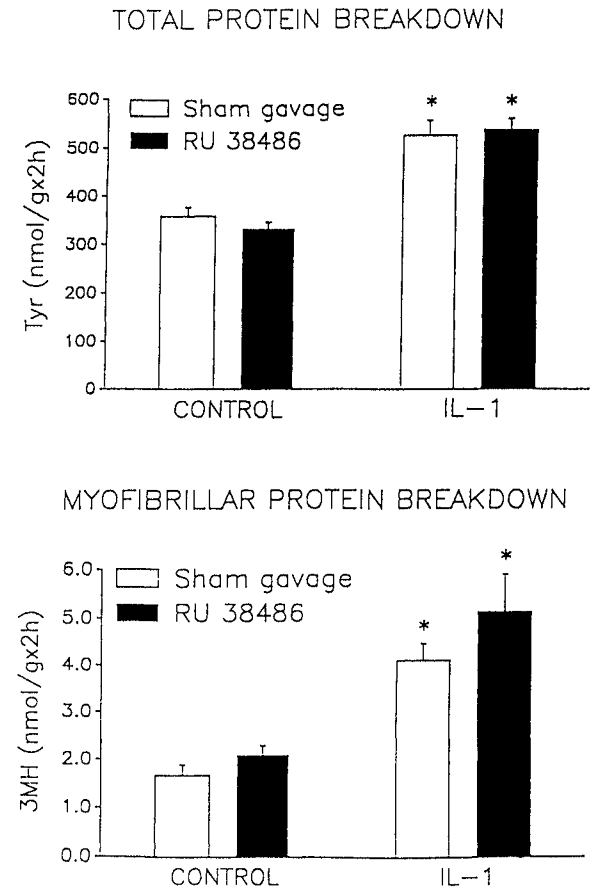

FIG. 1. The effect of rIL-1α (100 $\mu g\ kg^{-1}$ bw × 3) on total (upper panel) and myofibrillar protein (lower panel) breakdown rates following treatment with RU 38486 (5 mg $kg^{-1}$ bw × 3) or sham gavage. $n = 13$–15 per group; *$p < 0.05$ *vs* the corresponding control group.

Following administration of rTNFα, total and myofibrillar protein breakdown rates were increased by approximately 40% and 60%, respectively (Fig. 2). This increase in protein breakdown rates was completely blocked by RU 38486 (Fig. 2).

Plasma corticosterone levels were increased 30 min after rIL-1α or rTNFα administration and remained elevated for at least 4 h (Fig. 3). The response of plasma corticosterone levels was similar to the two cytokines.

### Discussion

The present results confirmed previous studies from this and other laboratories showing that both IL-1α[4] and TNFα[2,3,16] can induce muscle proteolysis when administered to experimental animals. The study also showed that the increase in muscle protein breakdown, induced by rIL-1α, was not affected by RU 38486, whereas TNF-induced muscle proteolysis was completely blocked by the glucocorticoid receptor antagonist. The results

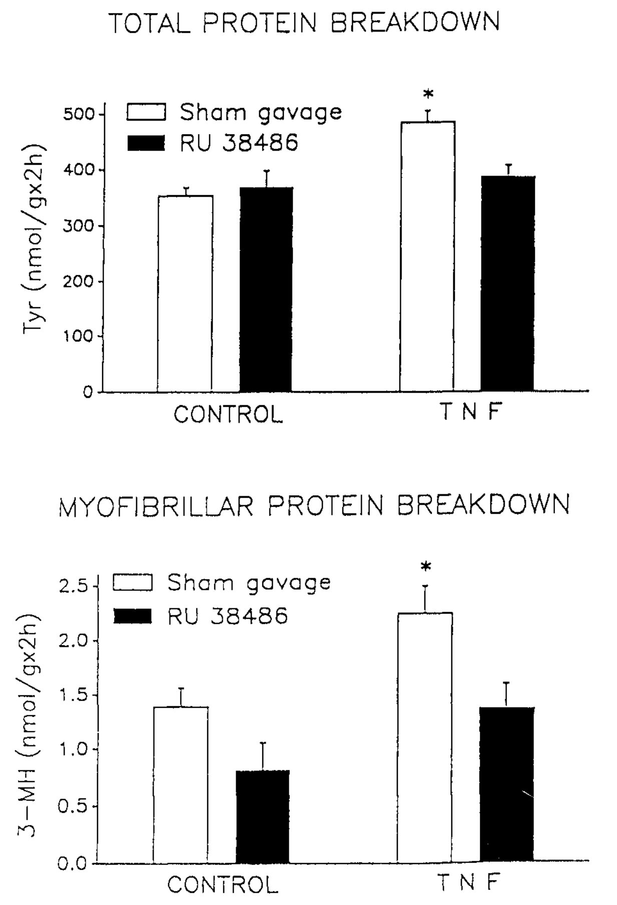

FIG. 2. The effect of rTNFα (100 μg kg$^{-1}$ bw × 3) on total (upper panel) and myofibrillar protein (lower panel) breakdown rates following treatment with RU 38486 (5 mg kg$^{-1}$ bw × 3) or sham gavage. $n$ = 11–13 per group; *$p < 0.05$ *vs* all other groups.

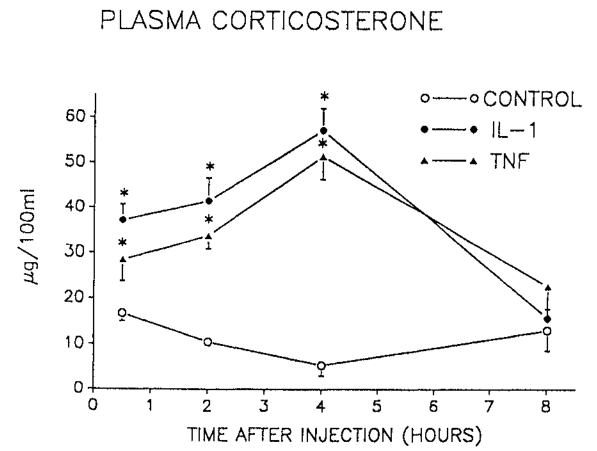

FIG. 3. Plasma corticosterone levels following administration of rIL-1α, rTNFα (100 μg kg$^{-1}$ bw) or control injection. $n$ = 4–5 per group. *$p < 0.05$ *vs* control.

suggest that TNF-induced muscle protein breakdown is mediated by glucocorticoids and that IL-1 stimulates muscle protein breakdown by other mechanism(s). Muscle protein synthesis was not determined in the present study because previous reports suggest that IL-1 and TNF do not regulate protein synthesis in skeletal muscle.[3,4]

RU 38486 is a potent glucocorticoid receptor antagonist with no agonist activity even at high concentrations.[13] The effectiveness of RU 38486 to block glucocorticoid receptors in different tissues, including skeletal muscle, was demonstrated previously.[17] At a dose of 5 mg kg$^{-1}$ body weight, RU 38486 blocked approximately 90% of glucocorticoid receptors in rat muscle 2 h after administration.[17] Chronic administration of the blocker inhibited dexamethasone-induced muscle atrophy.[17] A dose of 15 mg kg$^{-1}$ body weight given over 16 h, identical to the protocol used in the present study, completely prevented the proteolytic effect in rat muscle seen following injection of 200 mg kg$^{-1}$ body weight of corticosterone.[4]

The present result of glucocorticoid independent stimulation of muscle proteolysis following administration of rIL-1α is in line with a recent study from our laboratory.[4] Because in other studies IL-1 did not increase muscle protein degradation when added directly to incubated muscles, even at high concentrations,[5–8] it is not likely that muscle proteolysis following the administration of IL-1 *in vivo* reflects a direct effect on muscle. It is not known at present by which mechanism(s) IL-1 stimulates protein degradation in skeletal muscle. It may be speculated that catabolic hormones other than glucocorticoids, fever, haemodynamic changes and other steroid independent effects induced by IL-1 may account for its metabolic effects in muscle tissue.

In contrast to IL-1, the current study suggests that TNF requires the action of glucocorticoids to induce muscle protein breakdown. These results are in line with previous reports in which TNF-induced muscle catabolism was prevented by adrenalectomy.[12,18] The effect of RU 38486 on muscle proteolysis following TNF has not been reported previously. It should be noted that although the present results suggest that TNF-induced muscle proteolysis is mediated by glucocorticoids, the results may also be consistent with the concept that glucocorticoids act as a co-factor to TNF. Indeed, previous studies, in which TNF was administered to rats alone or in combination with corticosterone, suggest that glucocorticoids are an important cofactor to the cytokine.[18] Regardless of whether glucocorticoids act as a cofactor or as a second mediator released by TNF, the present study confirms that glucocorticoids are required for TNF to induce muscle protein breakdown.

Although the present results suggest that IL-1 and TNF stimulate muscle proteolysis through different mechanisms, other explanations need to be considered as well. For example, if IL-1 resulted in a more prominent release of glucocorticoids than TNF, the dose of RU 38486 may have been insufficient to block the effect of IL-1. Although plasma corticosterone levels were almost identical following a single injection of rIL-1α or rTNFα in the present study, it should be noted that plasma hormone levels may be different following repeated injections of the cytokines. In a recent study, mice became refractory to induction of serum corticosterone following repeated injections of TNF but not following repeated injections of IL-1.[19]

Because previous studies, both *in vivo* and *in vitro*, have demonstrated that TNF stimulates the release of IL-1 from macrophages and endothelial cells,[20,21] it is possible that the increased muscle protein breakdown, observed in the current experiments, was the result of IL-1 even when rats were treated with TNF and that TNF-induced release of IL-1 was mediated by glucocorticoids. Further experiments are required to resolve this issue. The interaction between IL-1, TNF and glucocorticoids is further complicated by the fact that exogenous, and possibly endogenous, glucocorticoids may affect cytokine production.[22–24]

The present results are important from a clinical standpoint because both IL-1 and TNF have been implicated as regulators of muscle protein breakdown during sepsis.[1,3,25] Sepsis results in increased degradation of muscle protein, in particular myofibrillar proteins,[26] similar to the response seen here following administration of IL-1 or TNF. Recent studies in our laboratory, using anti-TNF antibodies[3] or IL-1 receptor antagonists (unpublished observations) in septic rats support the concept that both cytokines are involved in the regulation of muscle protein breakdown during sepsis. When septic rats were treated with RU 38486, the increase in muscle protein breakdown was blunted but not completely blocked,[27] suggesting that sepsis-induced muscle proteolysis is mediated both by glucocorticoid dependent and glucocorticoid independent pathways. From the results in the present study it may be speculated that during sepsis glucocorticoids mediate the effect of TNF, whereas IL-1 stimulates muscle proteolysis through a glucocorticoid independent pathway.

### References

1. Baracos V, Rodemann HP, Dinarello CA, Goldberg AL. Stimulation of muscle protein degradation and prostaglandin $E_2$ release by leukocyte pyrogen (interleukin-1). *N Engl J Med* 1983; **308**: 553–558.
2. Flores EA, Bistrian BR, Pomposelli JJ, Dinarello CA, Blackburn GG, and Istfan NW. Infusion of tumour necrosis factor/cachectin promotes muscle catabolism in the rat. A synergistic effect with interleukin-1. *J Clin Invest* 1989; **83**: 1614–1622.
3. Zamir O, Hasselgren PO, Kunkel SL, Frederick JA, Higashiguchi T, Fischer JE. Evidence that tumour necrosis factor participates in the regulation of muscle proteolysis during sepsis. *Arch Surg* 1992; **127**: 170–174.
4. Zamir O, Hasselgren PO, von Allmen D, Fischer JE. The effect of interleukin-1α and the glucocorticoid receptor blocker RU 38486 on total and myofibrillar protein breakdown in skeletal muscle. *J Surg Res* 1991; **50**: 579–583.
5. Goldberg AL, Kettlehut IC, Furano K, Fagan JM, Baracos V. Activation of protein breakdown and prostaglandin $E_2$ production in rat skeletal muscle in fever is signalled by a macrophage product distinct from interleukin-1 or other known monokines. *J Clin Invest* 1988; **81**: 1378–1383.
6. Moldawer LL, Svaninger G, Gelin J, Lundholm KG. Interleukin-1 and tumour necrosis factor do not regulate protein balance in skeletal muscle. *Am J Physiol* 1987; **253**: C766–C773.
7. Hummel RP, Warner BW, Pedersen P, *et al. In vitro* effect of TNF, IL-1 and other monokines on skeletal muscle amino acid uptake and protein degradation. *Surg Forum* 1987; **38**: 13–15.
8. Hasselgren PO, James JH, Benson DW, Li S, Fischer JE. Is there a circulating proteolysis-inducing factor during sepsis? *Arch Surg* 1990; **125**: 510–514.
9. Tracey KJ, Lowry SF, Fahey TJ, *et al.* Cachectin/tumour necrosis factor induces lethal shock and stress hormone responses in the dog. *Surg Gynecol Obstet* 1987; **164**: 415–422.
10. Warren RS, Starnes HF, Alcock N, Calvano S, Brennan MD. Hormonal and metabolic response to recombinant human tumour necrosis factor in rat; *in vitro* and *in vivo. Am J Physiol* 1988; **255**: E206–E212.
11. Gwosdow AR, Kumar MSA, Bode HH. Interleukin-1 stimulation of hypothalamic–pituitary–adrenal axis. *Am J Physiol* 1990; **258**: E65–E70.
12. Mealy K, van Lanschot JJB, Robinson BG, Ramos J, Wilmore DW. Are the effects of tumour necrosis factor mediated by glucocorticoids? *Arch Surg* 1990; **125**: 42–48.
13. Philibert D. RU 38486: an original multi-faceted and antihormone *in vivo.* In: Agarwal MK, ed. *Adrenal Steroid Antagonism.* Berlin, New York: Walter de Gruyter & Co., 1984, 77–100.
14. Hasselgren PO, Hall-Angerås M, Angerås U, Benson D, James JH, Fischer JE. Regulation of total and myofibrillar protein breakdown in rat extensor digitorum longus and soleus muscle incubated flaccid or at resting length. *Biochem J* 1990; **267**: 37–44.
15. Gwosdow-Cohen A, Chen CL, Besch EL. Radioimmunoassay (RIA) of serum corticosterone in rats. *Proc Soc Exp Biol Med* 1982; **170**: 29–34.
16. Goodman MN. Tumour necrosis factor induces skeletal muscle protein breakdown in rats. *Am J Physiol* 1991; **260**: E727–E730.
17. Konagaya M, Bernard PA, Max SR. Blockade of glucocorticoid receptor binding and inhibition of dexamethasone-induced muscle atrophy in the rat by RU 38486, a potent glucocorticoid antagonist. *Endocrinology* 1986; **119**: 375–380.
18. Hall-Angerås M, Angerås U, Zamir O, Hasselgren PO, Fischer JE. Interaction between corticosterone and tumour necrosis factor stimulated protein breakdown in rat skeletal muscle, similar to sepsis. *Surgery* 1990; **108**: 460–466.
19. Mengozzi M, Ghezzi P. Defective tolerance to the toxic and metabolic effects of interleukin 1. *Endocrinology* 1991; **128**: 1668–1672.
20. Nawroth PP, Band I, Handley D, Cassimeris J, Chess L, Stern D. Tumour necrosis factor/cachectin interacts with endothelial cell receptors to induce release of interleukin-1. *J Exp Med* 1986; **163**: 1363–1375.
21. Dinarello CA, Cannon JG, Wolff SM, *et al.* Tumour necrosis factor (cachectin) is an endogenous pyrogen and induces production of interleukin-1. *J Exp Med* 1986; **163**: 1433–1450.
22. Strauch MJ, Wood DD. Reduction of serum interleukin-like activity after treatment with dexamethasone. *J Leuk Biol* 1985; **37**: 193–207.
23. Kern JA, Lamb RJ, Reed JC, Daniele RP, Nowell PC. Dexamethasone inhibition of interleukin-1β production by human monocytes. *J Clin Invest* 1988; **81**: 237–244.
24. Zuckerman SH, Shellhaas J, Butler LD. Differential regulation of lipopolysaccharide-induced interleukin-1 and tumour necrosis factor synthesis: effects of endogenous and exogenous glucocorticoids and the role of the pituitary–adrenal axis. *Eur J Immunol* 1989; **19**: 301–305.
25. Fong Y, Moldawer LL, Marano M, *et al.* Cachectin/TNF or IL-1α induces cachexia with redistribution of body proteins. *Am J Physiol* 1989; **256**: R659–R665.
26. Hasselgren PO, James JH, Benson DW, *et al.* Total and myofibrillar protein breakdown in different types of rat skeletal muscle: effect of sepsis and regulation by insulin. *Metabolism* 1989; **38**: 634–640.
27. Hall-Angerås M, Angerås U, Zamir O, Hasselgren PO, Fischer JE. Effect of the glucocorticoid receptor antagonist RU 38486 on muscle protein breakdown in sepsis. *Surgery* 1991; **109**: 468–473.

ACKNOWLEDGEMENTS. Supported in part by NIH grant DK37908-04 and by a grant from the Shriners of North America (#15861)

**Received 27 April 1992;**
**accepted in revised form 10 June 1992**
